# Cionin, a vertebrate cholecystokinin/gastrin homolog, induces ovulation in the ascidian *Ciona intestinalis* type A

**DOI:** 10.1038/s41598-021-90295-3

**Published:** 2021-05-25

**Authors:** Tomohiro Osugi, Natsuko Miyasaka, Akira Shiraishi, Shin Matsubara, Honoo Satake

**Affiliations:** Division of Integrative Biomolecular Function, Suntory Foundation for Life SciencesBioorganic Research Institute, 8-1-1 Seikadai, Seika-cho, Soraku-gun, Kyoto, 619-0284 Japan

**Keywords:** Cell growth, Evolutionary developmental biology, Peptides, Hormone receptors

## Abstract

Cionin is a homolog of vertebrate cholecystokinin/gastrin that has been identified in the ascidian *Ciona intestinalis* type A. The phylogenetic position of ascidians as the closest living relatives of vertebrates suggests that cionin can provide clues to the evolution of endocrine/neuroendocrine systems throughout chordates. Here, we show the biological role of cionin in the regulation of ovulation. In situ hybridization demonstrated that the mRNA of the cionin receptor, *Cior2*, was expressed specifically in the inner follicular cells of pre-ovulatory follicles in the *Ciona* ovary. Cionin was found to significantly stimulate ovulation after 24-h incubation. Transcriptome and subsequent Real-time PCR analyses confirmed that the expression levels of receptor tyrosine kinase (RTK) signaling genes and a matrix metalloproteinase (MMP) gene were significantly elevated in the cionin-treated follicles. Of particular interest is that an RTK inhibitor and MMP inhibitor markedly suppressed the stimulatory effect of cionin on ovulation. Furthermore, inhibition of RTK signaling reduced the MMP gene expression in the cionin-treated follicles. These results provide evidence that cionin induces ovulation by stimulating MMP gene expression via the RTK signaling pathway. This is the first report on the endogenous roles of cionin and the induction of ovulation by cholecystokinin/gastrin family peptides in an organism.

## Introduction

Ascidians are the closest living relatives of vertebrates in the Chordata superphylum, and thus they provide important insights into the evolution of peptidergic systems in chordates. Over the past two decades, a wide variety of neuropeptides and their receptors have been identified in the neural complex of *Ciona intestinalis* type A (synonym for *Ciona robusta*)^1^ including cionin, gonadotropin-releasing hormones (GnRHs), tachykinin (CiTK), calcitonin, vasopressin (CiVP), and insulin-like peptide^2–10^. Peptidomic analyses of the neural complex of *Ciona* have also identified more than 30 neuropeptides and peptide hormones, including various *Ciona*-specific peptides as well as homologous peptides^11,12^. Furthermore, our recent machine learning-based approach elucidated receptors for galanin-like peptide and 11 novel (*Ciona*-specific) neuropeptides^13^. In contrast, sexual steroidogenesis and their receptor genes have been lost in the *Ciona* genome during the evolution of chordates^14^, suggesting that neuropeptides are major factors for the biological regulation in *Ciona*. Interestingly, our recent anatomical analyses of transgenic *Ciona* revealed diverse innervation of peptidergic neurons of the central nervous system to peripheral organs including ovaries, strongly suggesting that the peptidergic system directly regulates ovarian follicle growth, maturation, and ovulation in *Ciona*^15,16^. Although a few *Ciona* neuropeptides such as CiTK, neurotensin-like peptide-6, and CiVP have been shown to participate in ovarian functions^11,17–19^, the biological roles of *Ciona* neuropeptides and the molecular mechanisms underlying the reproductive process largely remain to be investigated.

Cholecystokinin (CCK) and gastrin are a vertebrate brain/gut peptide and a gastric hormone, respectively^20^. These peptides possess a signature amino acid, a sulfated tyrosine, at positions 7 and 6 from the C-terminus, and harbor a common C-terminal amidated tetrapeptide motif (Trp-Met-Asp-Phe-NH_2_) that is essential for receptor activation^20^. Synteny analysis has suggested that CCK and gastrin genes might have arisen from a common ancestral gene through a first-round whole-genome duplication event during early vertebrate evolution^20^. A *Ciona* homolog of CCK, cionin, has been isolated from the *Ciona* neural complex and shares the C-terminal consensus motif of vertebrate CCK and gastrin^2,21^ Furthermore, cionin possesses two sulfated tyrosines at positions 6 and 7 from the C-terminus^2^. In addition, combined with the crucial phylogenetic position of *Ciona* as the sister group of vertebrates, the lack of a whole-genome duplication event during the evolution of *Ciona*^22^ suggests that cionin conserves common ancestral features of CCK and gastrin.

To date, two cognate cionin receptors, CioR1 and CioR2, have been identified^23,24^. These receptors show high sequence homology to vertebrate CCK/gastrin receptors (CCK1R and CCK2R). CioR1 and CioR2 trigger intracellular calcium mobilization in response to cionin^23,24^, suggesting that these receptors are G protein-coupled receptors (GPCRs) coupled with G_q_ protein. Molecular phylogenetic analysis demonstrated that both *Cior1* and *Cior2* genes are orthologous to the vertebrate CCKRs and were generated in the *Ciona*-specific evolutionary lineage^24^. *Cior1* and *Cior2* mRNAs are expressed in the neural complex, digestive organs, oral siphon, atrial siphon, and ovary^24^. Notably, *Cior2* mRNA is highly expressed specifically in the *Ciona* ovary, suggesting that cionin plays pivotal roles in reproductive control^24^.

In this study, we unraveled the biological functions of cionin. The present data indicated that cionin stimulates ovulation via activation of receptor tyrosine kinase (RTK) signaling pathways leading to upregulation of the expression of a matrix metalloproteinase (MMP) gene. The present study reveals a novel biological role for CCK/gastrin family peptides in the ovary, and provides clues to the evolution of the regulatory system of ovulation in chordates.

## Materials and methods

### Animals

Adult *Ciona intestinalis* type A (synonym for *Ciona robusta*)^1^ were cultivated at the Maizuru Fisheries Research Station of Kyoto University or Misaki Marine Biological Station of the University of Tokyo and maintained in sterile artificial sea water at 18 °C.

### In situ hybridization

The 3'-terminal region of *Cior2* (nt 1357–1662; GenBank accession numbers AB669183 and AB863059) was inserted into the pGEM-T easy vector (Promega, Madison, WI, USA). The plasmid was linearized and used with the digoxigenin-labeled RNA labeling kit (Roche Applied Science, Penzberg, Germany). The *Ciona* ovaries were dissected and fixed in 4% paraformaldehyde in 0.1 M phosphate buffer at 4 °C overnight. The fixed tissues were soaked in a refrigerated sucrose solution (30% sucrose in PBS) until they sank. They were embedded in Super Cryoembedding Medium-L1 (Leica Microsystems Japan, Tokyo, Japan) and sectioned at 10-µm thickness with a CryoStar NX70 cryostat (Thermo Fisher Scientific Inc., Waltham, MA, USA) at ‒20 °C. The sections were placed onto MAS-coated slides (Matsunami Glass Ind., Ltd., Osaka, Japan). Hybridization, washing, and detection were carried out as previously reported^4,25,26^. No positive signals were observed when sense probes were used, confirming the specificity of hybridization. The primer set used for the *Cior2* probe is listed in Table 1.

**Table 1:** Primer sets used in this study.

Gene name	Accession no	Forward	Reverse
**Probes for ISH**			
*Cior2*	AB669183/AB863059	GTGCTTCTACATCTTGTGCG	GTGCGCATTTTTTTATTTCG
**Real-time PCR**			
*Rora*	KH.C8.101/ KY.Chr8.673	GGAAAACACCCCAGGTCGAT	CTCTTCCAGGGAGCGTTTGT
*Fcol1*	KH.C7.633/ KY.Chr7.425	TCCCGTTCACCAATCAGAGC	GTGCACGCGTTTATAGCAGG
*Gla3b*	KH.C10.8/ KY.Chr10.1028	CCCCATGCAACGATATCGGA	TTCTGCTGGCACTCGTCAAT
*CiMmp2/9/13*	KH.L76.4/ KY.Chr3.680	CGTTGATGCTGCGATGGAAG	CCACTCGTGTTCTGCATCCT

Genbank accession number is referred to for *Cior2*. *Ciona* Ghost Databases (http://ghost.zool.kyoto-u.ac.jp/cgi-bin/gb2/gbrowse/kh/ and http://ghost.zool.kyoto-u.ac.jp/default_ht.html) are referred to for the accession numbers for *Rora*, *Fcol1*, *Gla3b*, and *CiMmp2/9/13.*

### Fractionation of *Ciona* follicles

Fractionation of *Ciona* follicles was performed as previously described^19,27^. In brief, approximately 8 *Ciona* ovaries were collected and washed with artificial sea water three times. Follicles were isolated from the ovaries and were collected into a normal tissue culture dish (Corning Inc., New York, USA). The isolated *Ciona* follicles were fractionated using a series of stainless-steel sieves of varying particle sizes (180, 150, 90, 63, 38, and 20 μm; TOKYO SCREEN CO. LTD., Tokyo, Japan). Follicles were collected from the 150-μm sieve and transferred to a cell culture dish with a cell-repellent surface (#628979; Greiner Bio-One GmbH, Frickenhausen, Germany). Immature/pre-ovulatory follicles (early stage II to late stage II follicles) were further isolated based on their size (average size 170–180 μm in diameter) under a stereomicroscope (Discovery V8, Carl Zeiss, Tokyo, Japan). The structure of the *Ciona* follicle is illustrated in Fig. S1.

### Evaluation of oocyte maturation and ovulation of cionin-treated follicles

Oocyte maturation and ovulation of *Ciona* follicles were evaluated in vitro as previously described^19,27^. In brief, 16–25 follicles of stage II were randomly allocated and incubated with or without ligands including cionin, and the respective rates of germinal vesicle break-down (GVBD) in the oocytes and rupture of the outer follicular cell layer were evaluated as an indicator of oocyte maturation and ovulation after a 24-h incubation. Synthetic cionin was prepared as previously described^24^ and used at a final concentration of 5 µM given that lower concentrations of other peptides are reportedly ineffective in *Ciona*^28^. Follicles incubated with artificial sea water were used as a control group. Follicles incubated with nonsulfated cionin (final concentration, 5 µM), which does not activate cionin receptors^24^, were used as a negative control group. Sunitinib malate^29,30^ (Cayman Chemical Company, MI) was used to inhibit a receptor tyrosine kinase (RTK) signaling based on the preliminary experiments (Table S1). MMP 2/9 inhibitor II^31^ (Calbiochem, San Diego, CA) was used to inhibit MMP activity based on our previous study^19^. Sunitinib malate stock solution (1 mM) was prepared in 100% dimethyl sulfoxide (DMSO) and diluted with artificial sea water at a final concentration of 1 µM. MMP2/9 inhibitor II solution (10 mM) was prepared in 100% DMSO and diluted with artificial sea water at a final concentration of 10 µM. Artificial sea water containing an equivalent concentration of DMSO (0.1%) was used as a control group in the inhibitor experiments.

### RNA-seq of cionin-treated follicles

Two independent sets of follicles were used for RNA-seq of the control group and cionin-treated group. Total RNA was extracted from 25 to 30 follicles of stage II using Sepasol-RNA I (Nacalai Tesque, Kyoto, Japan). The total RNA was further purified and treated with TURBO DNase as previously described^19,27^. A total of 200 ng of quality-verified RNA from each sample was subjected to RNA-seq analysis using a HiSeq1500 instrument (Illumina, San Diego, CA) in rapid mode as reported previously^32^. The resultant reads were aligned to the *Ciona* genes (KH, ver. 2013), which were downloaded from the Ghost Database (http://ghost.zool.kyoto-u.ac.jp/cgi-bin/gb2/gbrowse/kh/). The new Ghost Database (http://ghost.zool.kyoto-u.ac.jp/default_ht.html) was also referred to for new accession numbers^33^. The expression level of each gene was calculated as the gene-specific reads per kilobase per million total reads (RPKM). Genes satisfying the condition of read number > 10 or RPKM value > 0.5 were selected to calculate the RPKM ratio. Genes satisfying the condition of RPKM^cionin^ value/RPKM^control^ value > 1.5 were defined as those upregulated in the cionin-treated follicles. Genes satisfying the condition of RPKM^cionin^ value/RPKM^control^ value < 0.67 were defined as those downregulated in the cionin-treated follicles. Genes satisfying the condition of 1.12 > RPKM^cionin^ value/RPKM^control^ value > 0.90 were defined as those unchanged in the cionin-treated follicles. Among these, 985 genes were selected for upregulated genes and downregulated genes, and 1970 genes were selected for unchanged genes for gene ontology (GO) analysis. Subsequently, putative genes for transcripts were annotated based on a homology search of the NCBI database under the condition of *e*-value < 10^–3^ using Blast2GO software (version 4.1.9) with default parameters^34^. GO analyses were performed using Blast2GO software, and GO categories satisfying enrich score > 1.3 for upregulated categories or enrich score < -1.3 for downregulated categories are selected and visualized with Cytoscape software as previously reported^32^.

### Real-time PCR analysis of cionin-treated follicles

Total RNA was extracted from 25 to 30 follicles of stage II as described above. A 300-ng aliquot of DNase-treated total RNA was used for first-strand cDNA synthesis. Real-time PCR was performed using a CFX96 Touch Real-Time PCR Detection System (Bio-Rad Laboratories, Inc., Hercules, CA) as previously described^19,32^ to validate the RNA-seq data. The primers used for real-time PCR analyses are listed in Table 1. Gene expression levels were normalized to the expression of the ubiquitin-associated domain containing one gene (*CiUbac1*, KH.L133.5 or KY.Chr12.25), which was found to be constitutively expressed in the follicles as previously described^19^.

### Statistical analysis

Results are shown as means ± SEM. Gaussian distribution of data were validated by Kolmogorov–Smirnov test. Data were analyzed by Student's *t* test or one-way ANOVA followed by Tukey's post hoc test. Differences were accepted as significant for *P* < 0.05 or *P* < 0.01.

## Results

### Localization of *Cior2* mRNA in the *Ciona* ovary

**Figure 1:**
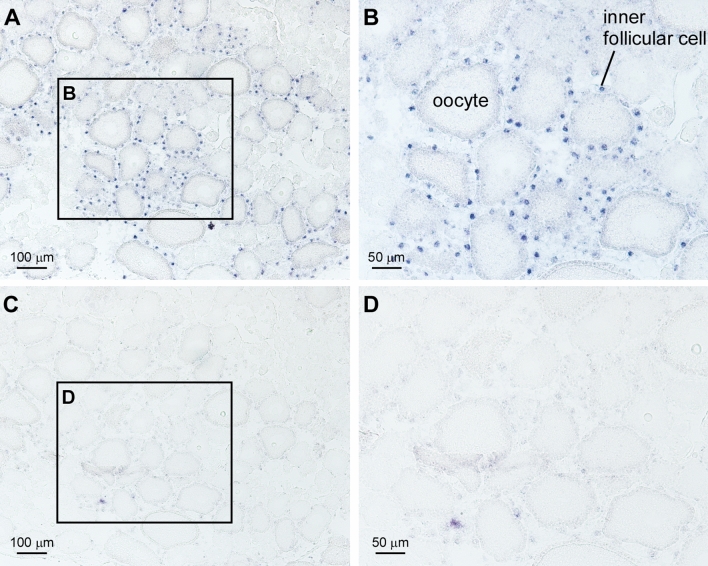
Localization of CioR2 mRNA in the ovary. **(A)** In situ hybridization of CioR2 mRNA in the ovary. Positive signals are shown in the inner follicular cells of stage II follicles. **(B)** Magnification of the rectangular region in **(A)**. **(C)** Negative control using the sense RNA probe. No hybridization signal was observed. **(D)** Magnification of the rectangular region in **(C)**.

To investigate the biological functions of cionin, we first analyzed the localization of cionin receptors in the ovary. *Cior2* mRNA was present exclusively in inner follicular cells, but not in oocytes or test cells, of pre-ovulatory follicles including stage II (Figs. 1, S1). These results suggest that the inner follicular cells of the pre-ovulatory follicles are major targets of cionin in the ovary. In addition, no positive signal was detected using sense probes (Fig. 1).

### Effects of cionin on the ovulation and maturation of follicles

**Figure 2:**
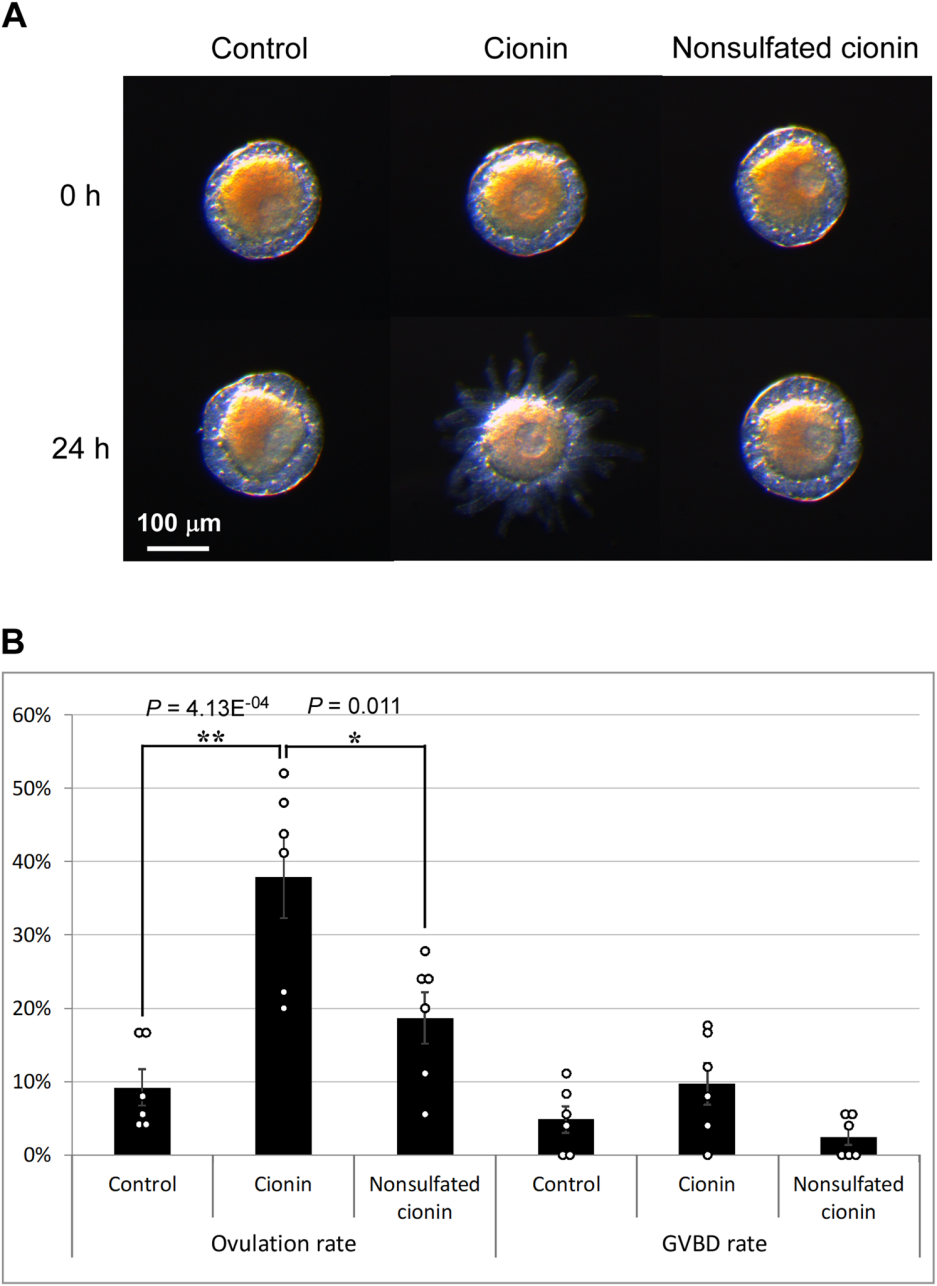
Effects of cionin on the ovulation and GVBD of follicles. **(A)** Representative images of follicles before and after treatment with 5 μM cionin or nonsulfated cionin for 24 h. Ovulation of stage II follicles was enhanced in the presence of cionin, whereas it was not in the presence of nonsulfated cionin. **(B)** Ovulation and GVBD rates were calculated after a 24-h incubation. Cionin significantly increased the ovulation rate but not the GVBD rate. Results are means ± SEM (n = 6). *, *P* < 0.05 cionin vs. nonsulfated cionin; and **, *P* < 0.01 control vs. cionin (one-way ANOVA followed by Tukey's post hoc test).

Pre-ovulatory follicles (average sizes of 170–180 μm in diameter), which were CioR2-positive (Fig. 1), were incubated with cionin for 24 h, and the ratios of ovulation and GVBD rates were estimated. The ovulation rate of cionin-treated follicles was significantly increased (9.2 ± 2.43% to 37.86 ± 5.51%), compared with the control follicles (Fig. 2, Supplemental Video, Supplemental data 1). Data of follicle assays were analyzed by one-way ANOVA followed by Tukey's post hoc test, and *P* values were obtained as 4.13E^-04^ for control vs cionin, 0.011 for cionin vs nonsulfated cionin, and 0.247 for control vs nonsulfated cionin (Fig. 2). In contrast, the GVBD rate of cionin-treated follicles was not significantly altered (Fig. 2 and Supplemental Video). Nonsulfated cionin, which fails to activate cionin receptors^24^, significantly increased neither ovulation nor GVBD rates after the 24-h incubation, proving that cionin specifically stimulated the ovulation (Fig. 2 and Supplemental Video, Supplemental data 1).

### Transcriptomic and real-time PCR analyses of cionin-treated follicles

**Figure 3:**
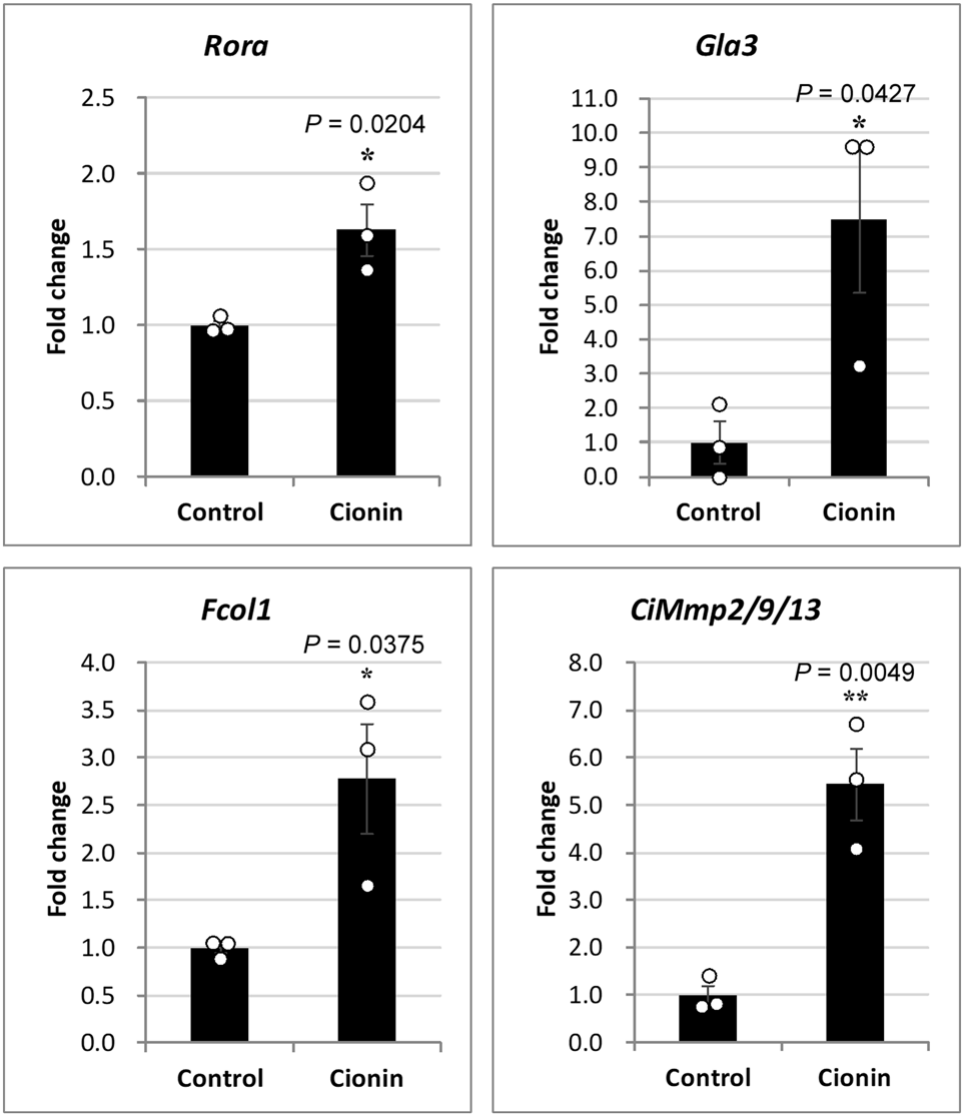
Real-time PCR analysis of cionin-treated follicles. Cionin stimulated the expression of *Rora*, *Gla3*, *Fcol1*, and *CiMmp2/9/13* genes after a 24-h incubation. Data from three independent experiments (n = 3) were analyzed by Student's *t* test and are shown as means ± SEM. *, *P* < 0.05; **, *P* < 0.01, control vs. cionin.

To investigate the mechanism underlying the cionin-induced ovulation, we performed RNA-seq and GO term analysis. RNA-seq for the control and cionin-treated groups yielded 12 million reads, respectively, for 101 single-end reads. Totals of 49,011 and 48,436 transcripts were mapped from reads of the control group and cionin-treated group, respectively. The resultant fastq files were deposited in the SRA database (SRA accession numbers: SRR10484994 and SRR10484993 for the control group, SRR10484992 and SRR10484991 for the cionin-treated group). GO term analysis of the fractionated follicles showed that cionin affected the expression of genes involved in the regulation of biological process, response to stimulus, locomotion, development/morphogenesis, single-organism cellular process, transport, and metabolic process in the category of "biological process" (Fig. S2A). In the category of "cellular component", membrane part, cell part, macromolecular complex, and organelle part were affected by cionin (Fig. S2B). In these categories, cytoskeletal part was downregulated, implying that cionin interacts suppressively with cytoskeletal proteins and induce morphological changes in ovulation. In the category of "molecular function", binding, catalytic activity, enzyme regulator activity, and transporter activity were affected by cionin (Fig. S2C). In these categories, peptidase activities are largely upregulated by cionin, implying that some peptidases that digest above mentioned cytoskeletal proteins are activated by cionin and resulted in ovulation. In addition, transmembrane proteins or protein kinases may participate in the regulation of ovulation induced by cionin. Among genes included in the above-mentioned GO categories, RTK-like orphan receptor (*Rora*), fibrillar collagen (*Fcol1*), transmembrane γ-carboxyglutamic acid protein 3 (*Gla3*), and MMP (*CiMmp2/9/13*) were considerably increased in the cionin-treated groups, compared with the control groups (Table S2). Real-time PCR further confirmed 1.6-fold, 2.8-fold, 7.5-fold, and 5.5-fold upregulation of the expressions of *Rora*, *Fcol1*, *Gla3*, and *CiMmp2/9/13* in the cionin-treated groups (Fig. 3). Data for real-time PCR were analyzed by Student's *t* test, and *P* values were obtained as 0.0204 for *Rora*, 0.0375 for *Fcol1*, 0.0427 for *Gla3*, and 0.0049 for *CiMmp2/9/13* (Fig. 3). The comparison between RNA-seq and real-time PCR data are shown in Fig. S3.

### Key roles for RTK and MMP signaling in ovulation by cionin

**Figure 4:**
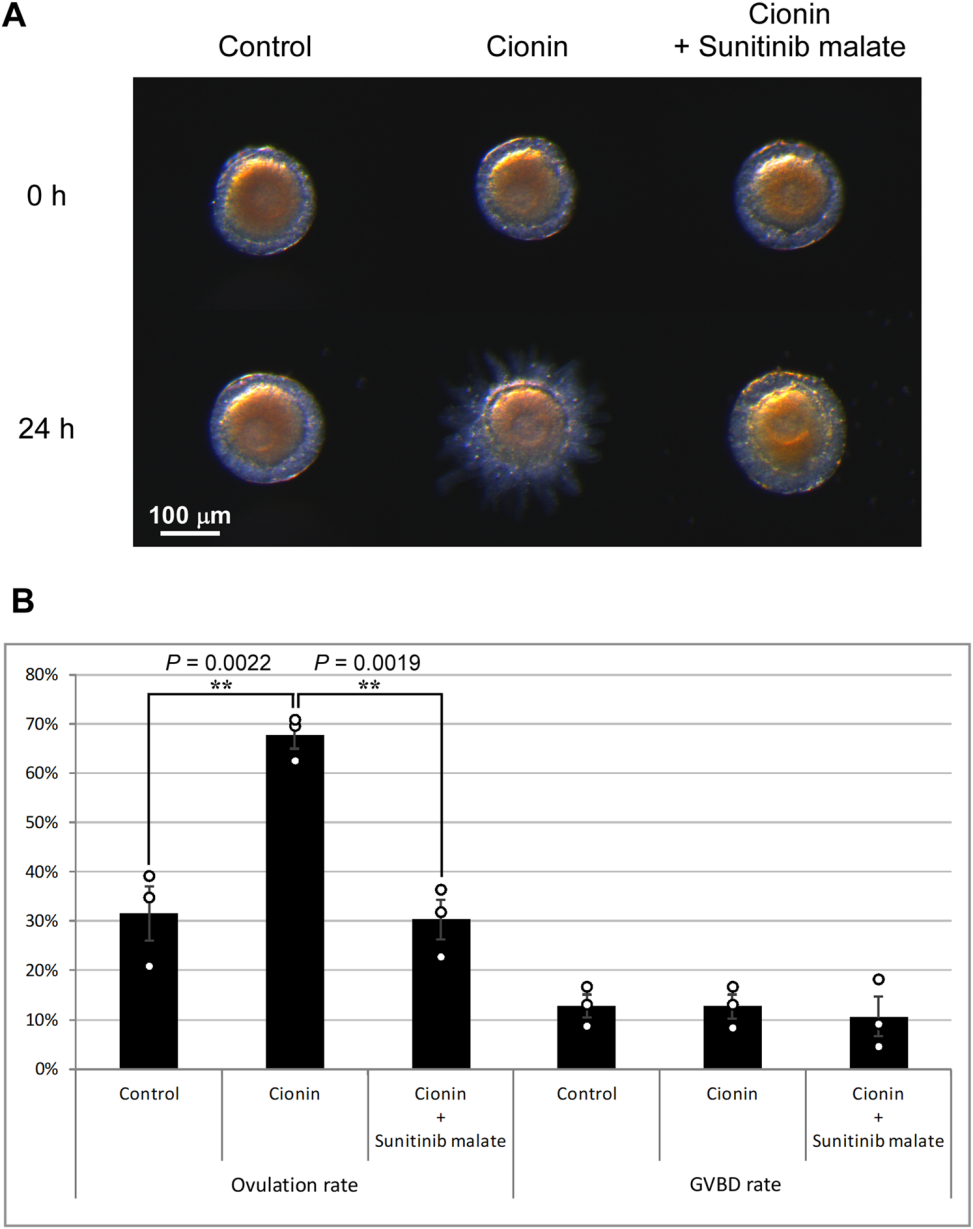
Effect of the receptor tyrosine kinase inhibitor, sunitinib malate, on the ovulation and GVBD of cionin-treated follicles. **(A)** Representative images of follicles before and after treatment with 5 μM cionin or 5 μM cionin and 1 mM sunitinib malate for 24 h. Cionin-induced ovulation of stage II follicles was disrupted by sunitinib malate. **(B)** Ovulation and GVBD rates were calculated after the 24-h incubation. Sunitinib malate significantly inhibited ovulation in the cionin-treated follicles. Sunitinib malate did not affect GVBD rate. Results are means ± SEM (n = 3). **, *P* < 0.01 control vs. cionin or cionin vs. cionin + sunitinib malate (one-way ANOVA followed by Tukey's post hoc test).

**Figure 5:**
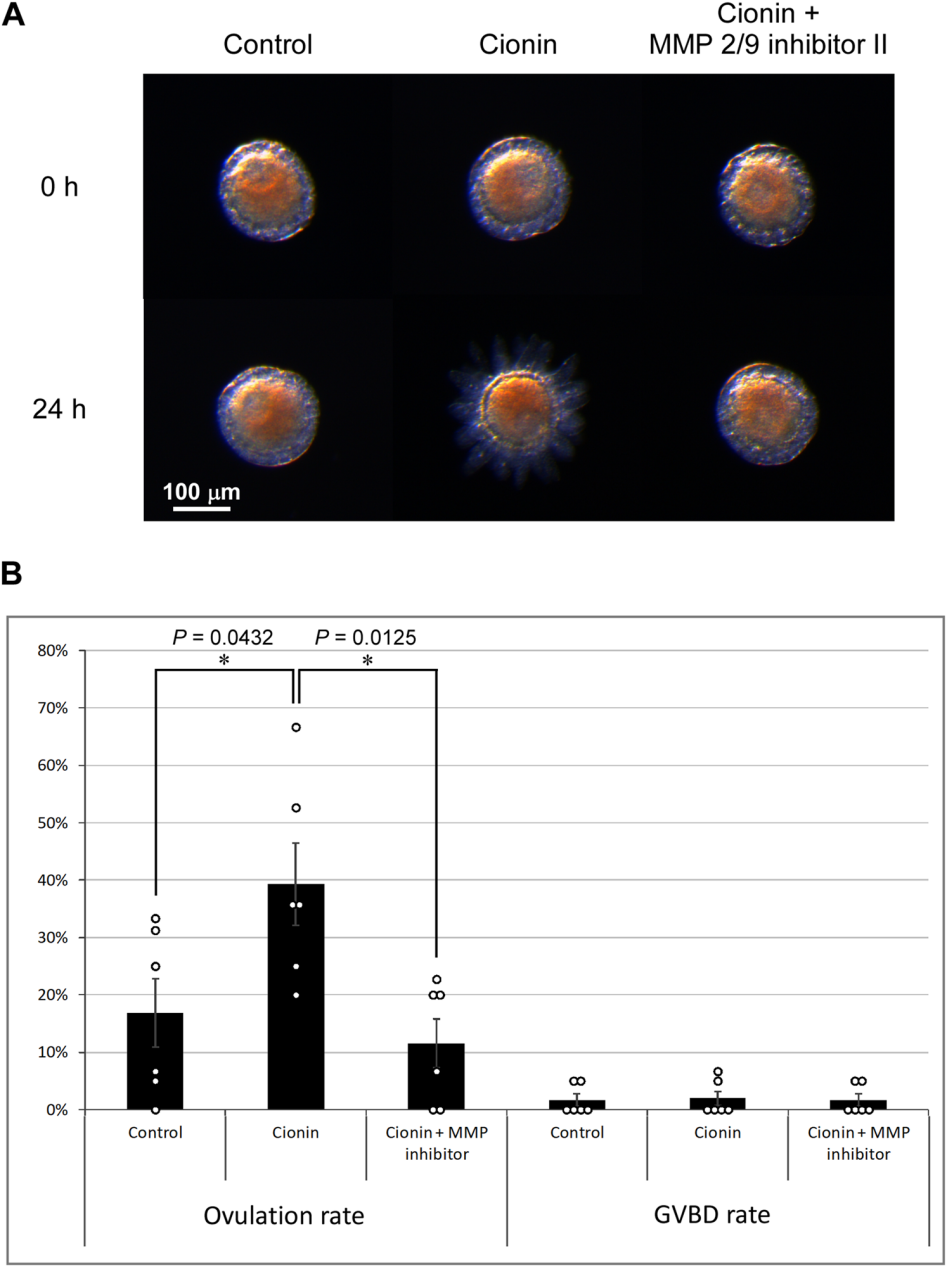
Effect of MMP2/9 inhibitor II on the ovulation and GVBD of cionin-treated follicles. **(A)** Representative images of follicles before and after treatment with 5 μM cionin or 5 μM cionin and 10 µM MMP2/9 inhibitor II for 24 h. Cionin-induced ovulation of stage II follicles was disrupted by MMP2/9 inhibitor II. **(B)** Ovulation and GVBD rates were calculated after the 24-h incubation. MMP2/9 inhibitor II significantly inhibited ovulation in the cionin-treated follicles. MMP2/9 inhibitor II did not affect GVBD rate. Results are means ± SEM (n = 6). *, *P* < 0.05 control vs. cionin or cionin vs. MMP2/9 inhibitor II + cionin (one-way ANOVA followed by Tukey's post hoc test).

**Figure 6:**
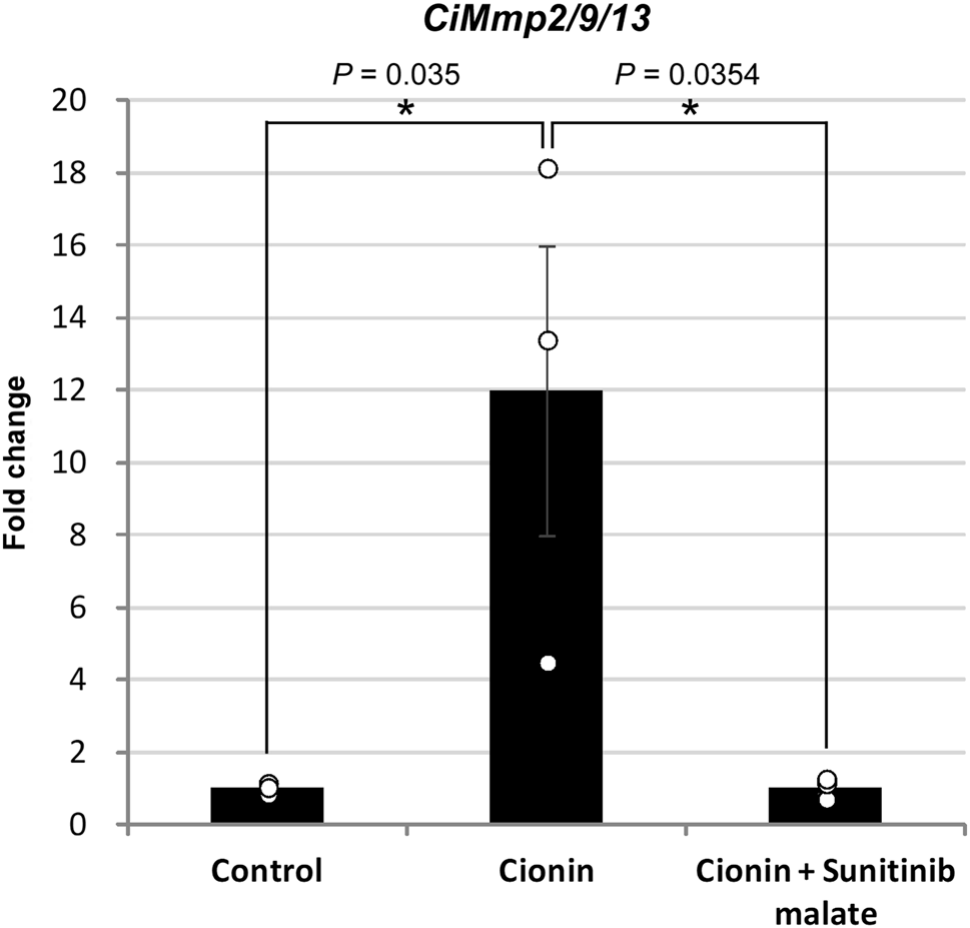
Real-time PCR analysis of cionin- and sunitinib malate-treated follicles. Sunitinib malate arrested the upregulation of the *CiMmp2/9/13* gene by cionin after a 24-h incubation. Results are means ± SEM (n = 3). *, *P* < 0.05 control vs. cionin or cionin vs. cionin + sunitinib malate (one-way ANOVA followed by Tukey's post hoc test).

**Figure 7:**
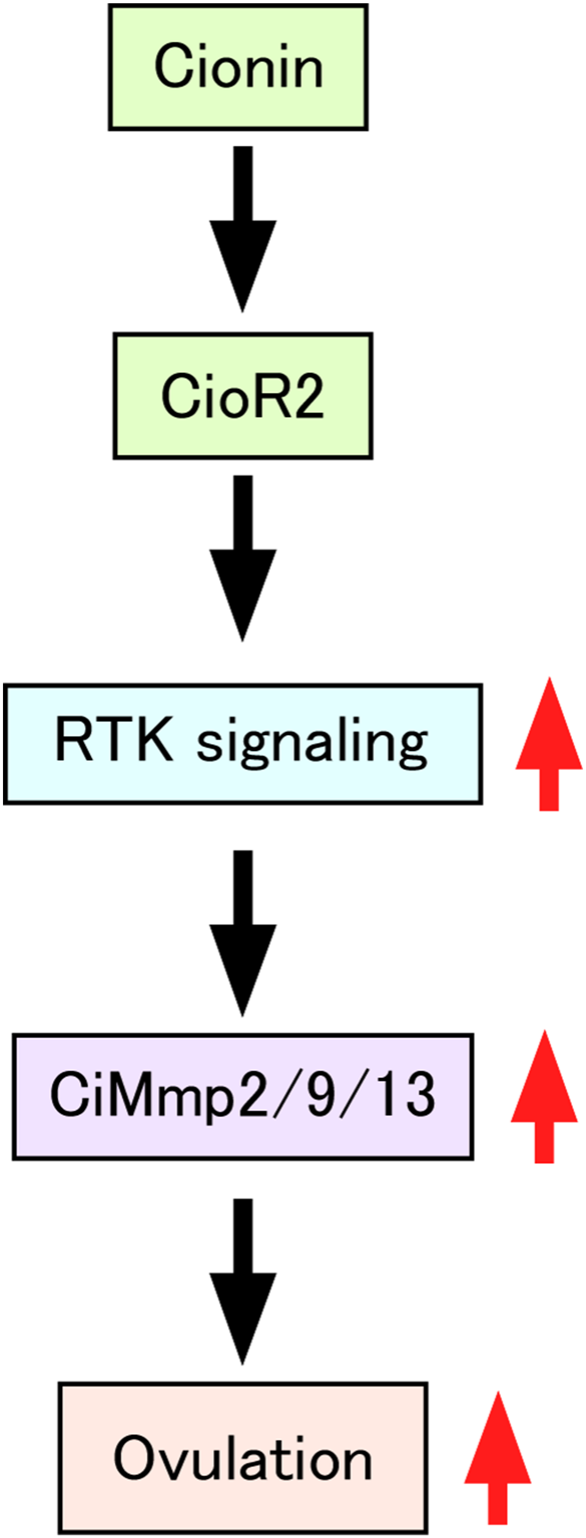
Schematic illustration of the regulatory mechanisms of cionin-directed ovulation. Cionin interacts with its receptor, CioR2, and activates RTK signaling. Subsequent induction of CiMmp2/9/13, which digests collagens in the outer follicular cell layer, leads to ovulation.

As shown in Fig. 3, cionin upregulated the expression of *Rora*, *Fcol1*, and *Gla3*, which are related to the RTK-signaling in mammals^38–41^. We thus examined whether the RTK signaling are involved in the cionin-induced ovulation. Of particular interest is that the ovulation rate of the cionin-treated follicles was markedly decreased by the RTK inhibitor, sunitinib malate, after the 24-h incubation (67.63 ± 2.59% to 30.3 ± 4.01%), whereas sunitinib malate did not affect the rate of GVBD (Fig. 4, Supplemental data 2). Data for the ovulation assays were analyzed by one-way ANOVA followed by Tukey's post hoc test, and *P* values were obtained as 0.0022 for control vs cionin, 0.0019 for cionin vs cionin + sunitinib malate, and 0.975 for control vs cionin + sunitinib malate (Fig. 4). These results indicated that RTK signaling participates in ovulation induced by cionin. Subsequently, we also examined the effect of an MMP inhibitor on the ovulation rate of cionin-treated follicles, given that cionin also significantly increased the expression of the *CiMmp2/9/13* gene (Fig. 3). As shown in Fig. 5, the ovulation of the cionin-treated follicles was significantly suppressed by MMP 2/9 inhibitor II after the 24-h incubation (39.29 ± 7.14% to 11.57 ± 4.31%). In contrast, MMP 2/9 inhibitor II did not alter the rate of GVBD (Fig. 5, Supplemental data 3). Data for the ovulation assays were analyzed by one-way ANOVA followed by Tukey's post hoc test, and *P* values were obtained as 0.0432 for control vs cionin, 0.0125 for cionin vs cionin + MMP 2/9 inhibitor II, and 0.804 for control vs cionin + MMP 2/9 inhibitor II (Fig. 5). In combination, we concluded that MMP2/9/13 was an important factor for cionin-induced ovulation. Furthermore, real-time PCR demonstrated that the administration of sunitinib malate arrested the upregulation of the *CiMmp2/9/13* gene by cionin, confirming that RTKs play an essential role in the upregulation of the expression of *CiMmp2/9/13* by cionin (Fig. 6). Data for real-time PCR were analyzed by one-way ANOVA followed by Tukey's post hoc test, and *P* values were obtained as 0.0350 for control vs cionin, 0.0354 for cionin vs cionin + sunitinib malate, and 1.00 for control vs cionin + sunitinib malate (Fig. 6). Altogether, these results verified that cionin induced ovulation via upregulation of the RTK-signaling-MMP activation pathway (Fig. 7).

## Discussion

CCK is a major vertebrate brain/gut peptide and plays multiple roles in the regulation of food intake, appetite, learning, and emotion^20^. In contrast, the endogenous role of the *Ciona* CCK homolog, cionin, has not been investigated. In the present study, we identified cionin as a inducer of ovulation and verified the underlying fundamental molecular mechanisms. The cionin gene is expressed almost exclusively in the cerebral neurons^2,12,24^, and its cognate receptor, CioR2^24^, was shown to be localized to the inner follicular cells of stage II follicles in the ovary (Fig. 1). Moreover, the *Ciona* ovary is innervated by multiple neuropeptidergic neurons in the cerebral ganglion^15^. Combined with these findings, the present study indicates that cionin is produced in and secreted from the cerebral ganglion to the ovary, and directly acts on inner follicular cells followed by the induction of ovulation (Fig. 2). To the best of our knowledge, this is the first evidence of a biological role of CCK family peptides in the regulation of ovulation in all animals.

Follicular growth, maturation, and ovulation are crucial processes in the development of an animal, and thus these reproductive processes are expected to be regulated by multiple signaling pathways involving various endogenous factors. In the present study, cionin was shown to activate ovulation via activation of CiMMP2/9/13 (Figs. 2, 5, Supplemental Video). Recently, we provided evidence that the *Ciona* VP homolog, CiVP, induces ovulation via the activation of CiMMP2/9/13^19^. In addition, MMPs are highly conserved collagenases that are known to play pivotal roles in ovulation in teleosts^19,39^, suggesting that the induction of gene expression and enzymatic activity of MMPs are evolutionarily conserved in the ovulation process of chordates. In contrast, there are differences between cionin and CiVP systems. First, *Cior2* was expressed in inner follicular cells (Fig. 1), whereas the CiVP receptor gene (*CiVpr*) is expressed in oocytes^19^. Second, cionin is responsible for only ovulation (Fig. 2, Supplemental Video), whereas CiVP participates in both oocyte maturation and ovulation^19^. As for the signaling pathway, cionin was shown to be involved in RTK signaling for the induction of CiMMP2/9/13 (Figs. 3, 4), whereas CiVP upregulates *CiMmp2/9/13 *via mitogen-activated protein kinase (MAPK) signaling pathways^19^. The differences in the signaling pathway between cionin and CiVP awaits further study, because MAPK was suggested to be regulated downstream of RTK signaling^40^. Altogether, the present study revealed that activation of CiMMP2/9/13 for ovulation is regulated by multiple endogenous factors and signaling pathways. The presence of such multiple regulatory systems also sheds light on the biological significance of CiMMP2/9/13 in ovulation in *Ciona*. The neuropeptides including cionin, CiTK, CiVP or other reagent including cAMP analog did not exhibit 100% effect in follicle growth, oocyte maturation, or ovulation in *Ciona* as well as other ascidians^17,19,41,42^. This is mainly due to the heterogenous quality of follicles. Low-quality follicles that are not competent in normal growth and maturation were included in the collected follicles. Moreover, it is impossible to discriminate high-quality follicles from low-ones during follicle collection. In addition, the expression of multiple neuropeptide receptors including CiTK receptor, CiVP receptor, cionin receptors in the oocyte or follicle cells suggests that the follicle growth, oocyte maturation and ovulation are regulated not only by one key factor but by multiple factors^17,19,24^.

A striking feature of the regulatory mechanism underlying *Ciona* ovulation is that RTK signaling factors participate in cionin-induced ovulation (Figs. 3, S2). RORa is a member of the RTK family in vertebrates^35^. GLA3 is a vertebrate transmembrane gamma-carboxyglutamic acid protein that has multiple epidermal growth factor (EGF) domains^36^ and is presumed to be a ligand of another RTK, Axl^36^. Fibrillar collagens including FCOL1 activate discoidin domain receptors (DDRs), which also belongs to the RTK family^37,38^. An inhibitor of multiple RTKs, sunitinib malate that inhibits DDRs^43^, ROR^44^, and Axl^5^ significantly inhibited the increase of the ovulation rate of cionin-treated follicles (Fig. 4), suggesting that RORa, GLA3, and FCOL1 are involved in the regulation of cionin-induced ovulation although the functions of these genes in *Ciona* await further study. In vertebrates, various GPCRs, including CCK receptors, activate RTKs via various signaling pathways including intracellular calcium, protein kinase C, and Src protein kinase via coupling with G_q_ protein^45–47^. These findings are compatible with the present study demonstrating that CioR2 stimulates RTK signaling, given that our in vitro study suggested that CioR2 couples with G_q_ protein^24^. Consequently, the present study provides evidence that cionin stimulates ovulation via upregulation of the CioR2-RTK signaling pathway in follicles (Fig. 7). It is also noteworthy that sunitinib malate inhibits ovulation but does not affect follicle growth or maturation in mice^48^. In addition, RTK signaling lies downstream of the luteinizing hormone surge and is essential to initiate the ovulatory response in mammals^49^. These results imply that RTKs are involved in ovulation in mammals, although no factors inducing RTK signaling in mammalian ovulation have been identified. In other words, CCK may also play some roles in ovulatory processes in mammals.

We also revealed upregulation of *CiMMP2/9/13 *via activation of RTK signaling, given that the cionin-induced *CiMMP2/9/13* gene expression was markedly suppressed by sunitinib malate (Fig. 5). *Ciona* possesses a total of seven MMP-like genes including *CiMMP2/9/13*^50^, and several MMPs may participate in the regulation of ovulation. This signaling in *Ciona* is compatible with previous findings indicating that MMPs are induced by RTKs, including discoidin domain receptors and vascular endothelial growth factor in mammalian smooth muscle cells or human placenta choriocarcinoma cell lines^51,52^. These in vitro studies imply that the RTK-MMP regulatory pathway also functions in the ovaries in mammals, although neither the proteases essential for mammalian ovulation nor the signaling molecules that upregulate the RTK pathways have been investigated. Moreover, the *Cck2r* gene is expressed in the ovaries of adult mice^53^ (BioProject accession number: PRJNA66167), and CCK2R has been reported to induce the expression of an RTK-related gene in rat gastric epithelial cells^54^. Altogether, these findings lead to a hypothesis that CCK/gastrin-RTK signaling is also responsible for the regulation of ovulation in vertebrates, including mammals, and that the ovulation mechanism regulated by the CCK family peptides-RTK signaling-MMP pathway, elucidated in the present study, is conserved during the evolution of chordates. The investigation of the biological roles of CCK/gastrin in ovulation or other ovarian functions in vertebrates is underway.

In conclusion, we have substantiated that the *Ciona* CCK homolog, cionin, induces ovulation by upregulating MMP via the RTK signaling pathway in *Ciona*, the closest relative of vertebrates. The present study has clarified not only the novel regulatory mechanisms underlying ovulation in *Ciona* and a novel biological role for CCK/gastrin family peptides in chordates, but also paved the way for understanding the biological roles and evolution of neuropeptidergic regulation of ovarian functions in chordates.

## Supplementary Information

Supplementary Video.

Supplementary Information

Supplementary Data 1.

Supplementary Data 2.

Supplementary Data 3.

## Data Availability

The transcriptome data are publicly available under accession numbers SRR10484994 and SRR10484993 for the control group, and SRR10484992 and SRR10484991 for the cionin-treated group in the NCBI SRA database.
