# Supplementary material for: Cionin, a vertebrate cholecystokinin/gastrin homolog, induces ovulation in the ascidian Ciona intestinalis type A
Source: Sci Rep. 2021 May 25;11:10911. doi: 10.1038/s41598-021-90295-3 (PMC8149874; doi:10.1038/s41598-021-90295-3)
Supplement: Supplementary file 2 — Supplementary Information [file 41598_2021_90295_MOESM2_ESM.pdf]

Supporting Information

Cionin, a vertebrate cholecystokinin/gastrin homolog, induces ovulation in the ascidian *Ciona intestinalis* Type A.

Tomohiro Osugi, Natsuko Miyasaka, Akira Shiraishi, Shin Matsubara, Honoo Satake*

Suntory Foundation for Life Sciences, Bioorganic Research Institute, Division of Integrative Biomolecular Function, 8-1-1 Seikadai, Seika-cho, Soraku-gun, Kyoto 619-0284, Japan

*Correspondence to: Honoo Satake, Suntory Foundation for Life Sciences, Bioorganic Research Institute, Division of Integrative Biomolecular Function, 8-1-1 Seikadai, Seika-cho, Soraku-gun, Kyoto 619-0284, Japan
E-mail: satake@sunbor.or.jp

Fig. S1. Schematic illustration of the *Ciona* follicle. The immature oocyte has a germinal vesicle (GV). The oocyte is surrounded by the inner follicular cells and outer follicular cells.

Fig. S2. Gene ontology (GO) enrichment analysis of increased and decreased genes in the cionin-treated follicles. The GO category shown is "biological process" in the Fig. S2A, "Cellular Component" in the Fig. S2B, and "Molecular Function" in the Fig. S2C. Each node represents a GO term. The circle size of each node indicates the number of genes with that GO term. The color of each node represents the enrichment score. Red and blue colored nodes indicate high enrichment scores of increased and decreased genes in cionin-treated follicles, respectively. Edges represent "is_a" connections between GO terms.

Fig. S3. Comparison between RNA-seq data and Real-time PCR data of *Rora*, *Fcol1*, *Gla3*, and *CiMmp2/9/13*. RNA-seq data are shown as reads per kilobase per million total reads (RPKM). Real-time PCR data are shown as fold change as in Fig. 3.

Supplemental video.

Cionin induces ovulation. Isolated stage II follicles were incubated with 5 μM cionin (left) or 5 μM nonsulfated cionin (right) for 24 h. Time-lapse images were captured every 5 min. Ovulation was observed in the cionin-treated follicle, whereas it was not with nonsulfated cionin treatment.

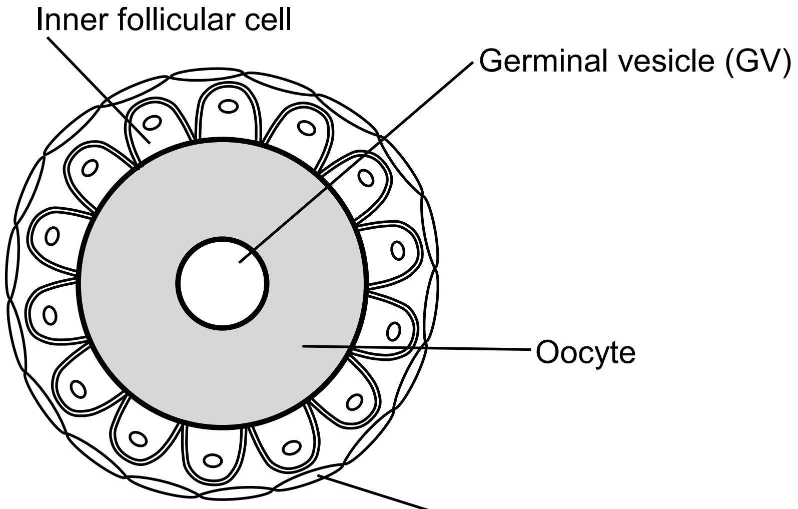
Inner follicular cell
Germinal vesicle (GV)
Oocyte
Outer follicular cell

Fig. S1

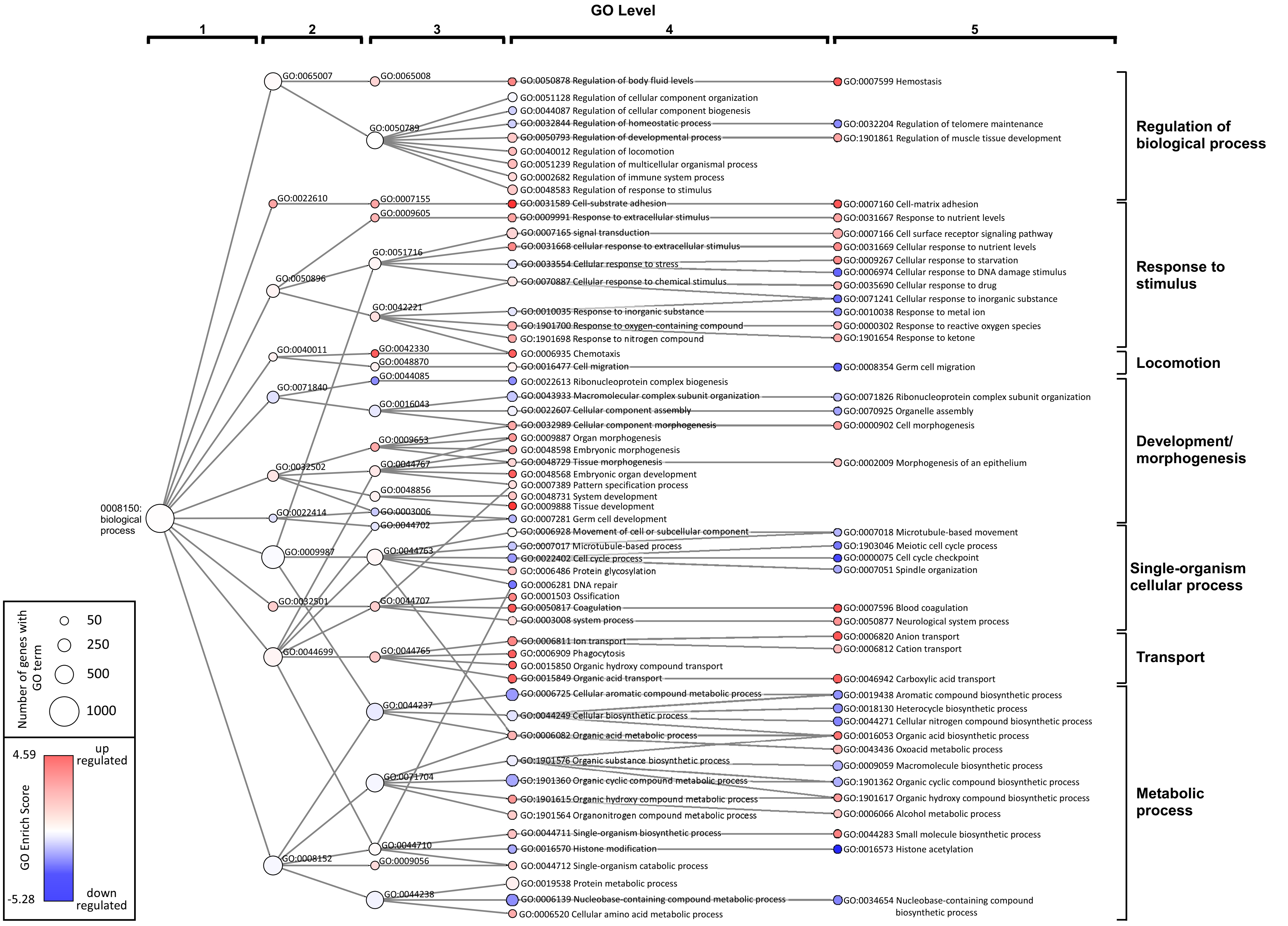

GO Level
1
2
3
4
5
0008150: biological process
GO:0065007
GO:0022610
GO:0050896
GO:0040011
GO:0071840
GO:0032502
GO:0022414
GO:0009987
GO:0032501
GO:0044699
GO:0008152
GO:0065008
GO:0050789
GO:0007155
GO:0009605
GO:0051716
GO:0042221
GO:0042330
GO:0048870
GO:0044085
GO:0016043
GO:0009653
GO:0044767
GO:0048856
GO:0003006
GO:0044702
GO:0044763
GO:0044707
GO:0044765
GO:0044237
GO:0071704
GO:0044710
GO:0009056
GO:0044238
GO:0050878 Regulation of body fluid levels
GO:0051128 Regulation of cellular component organization
GO:0044087 Regulation of cellular component biogenesis
GO:0032844 Regulation of homeostatic process
GO:0050793 Regulation of developmental process
GO:0040012 Regulation of locomotion
GO:0051239 Regulation of multicellular organismal process
GO:0002682 Regulation of immune system process
GO:0048583 Regulation of response to stimulus
GO:0031589 Cell-substrate adhesion
GO:0009991 Response to extracellular stimulus
GO:0007165 signal transduction
GO:0031668 cellular response to extracellular stimulus
GO:0033554 Cellular response to stress
GO:0070887 Cellular response to chemical stimulus
GO:0010035 Response to inorganic substance
GO:1901700 Response to oxygen-containing compound
GO:1901698 Response to nitrogen compound
GO:0006935 Chemotaxis
GO:0016477 Cell migration
GO:0022613 Ribonucleoprotein complex biogenesis
GO:0043933 Macromolecular complex subunit organization
GO:0022607 Cellular component assembly
GO:0032989 Cellular component morphogenesis
GO:0009887 Organ morphogenesis
GO:0048598 Embryonic morphogenesis
GO:0048729 Tissue morphogenesis
GO:0048568 Embryonic organ development
GO:0007389 Pattern specification process
GO:0048731 System development
GO:0009888 Tissue development
GO:0007281 Germ cell development
GO:0006928 Movement of cell or subcellular component
GO:0007017 Microtubule-based process
GO:0022402 Cell cycle process
GO:0006486 Protein glycosylation
GO:0006281 DNA repair
GO:0001503 Ossification
GO:0050817 Coagulation
GO:0003008 system process
GO:0006811 Ion transport
GO:0006909 Phagocytosis
GO:0015850 Organic hydroxy compound transport
GO:0015849 Organic acid transport
GO:0006725 Cellular aromatic compound metabolic process
GO:0044249 Cellular biosynthetic process
GO:0006082 Organic acid metabolic process
GO:1901576 Organic substance biosynthetic process
GO:1901360 Organic cyclic compound metabolic process
GO:1901615 Organic hydroxy compound metabolic process
GO:1901564 Organonitrogen compound metabolic process
GO:0044711 Single-organism biosynthetic process
GO:0016570 Histone modification
GO:0044712 Single-organism catabolic process
GO:0019538 Protein metabolic process
GO:0006139 Nucleobase-containing compound metabolic process
GO:0006520 Cellular amino acid metabolic process
GO:0007599 Hemostasis
GO:0032204 Regulation of telomere maintenance
GO:1901861 Regulation of muscle tissue development
GO:0007160 Cell-matrix adhesion
GO:0031667 Response to nutrient levels
GO:0007166 Cell surface receptor signaling pathway
GO:0031669 Cellular response to nutrient levels
GO:0009267 Cellular response to starvation
GO:0006974 Cellular response to DNA damage stimulus
GO:0035690 Cellular response to drug
GO:0071241 Cellular response to inorganic substance
GO:0010038 Response to metal ion
GO:0000302 Response to reactive oxygen species
GO:1901654 Response to ketone
GO:0008354 Germ cell migration
GO:0071826 Ribonucleoprotein complex subunit organization
GO:0070925 Organelle assembly
GO:0000902 Cell morphogenesis
GO:0002009 Morphogenesis of an epithelium
GO:0007018 Microtubule-based movement
GO:1903046 Meiotic cell cycle process
GO:0000075 Cell cycle checkpoint
GO:0007051 Spindle organization
GO:0007596 Blood coagulation
GO:0050877 Neurological system process
GO:0006820 Anion transport
GO:0006812 Cation transport
GO:0046942 Carboxylic acid transport
GO:0019438 Aromatic compound biosynthetic process
GO:0018130 Heterocycle biosynthetic process
GO:0044271 Cellular nitrogen compound biosynthetic process
GO:0016053 Organic acid biosynthetic process
GO:0043436 Oxoacid metabolic process
GO:0009059 Macromolecule biosynthetic process
GO:1901362 Organic cyclic compound biosynthetic process
GO:1901617 Organic hydroxy compound biosynthetic process
GO:0006066 Alcohol metabolic process
GO:0044283 Small molecule biosynthetic process
GO:0016573 Histone acetylation
GO:0034654 Nucleobase-containing compound biosynthetic process
Regulation of biological process
Response to stimulus
Locomotion
Development/ morphogenesis
Single-organism cellular process
Transport
Metabolic process
Number of genes with GO term
50
250
500
1000
GO Enrich Score
4.59
-5.28
up regulated
down regulated

Fig. S2A

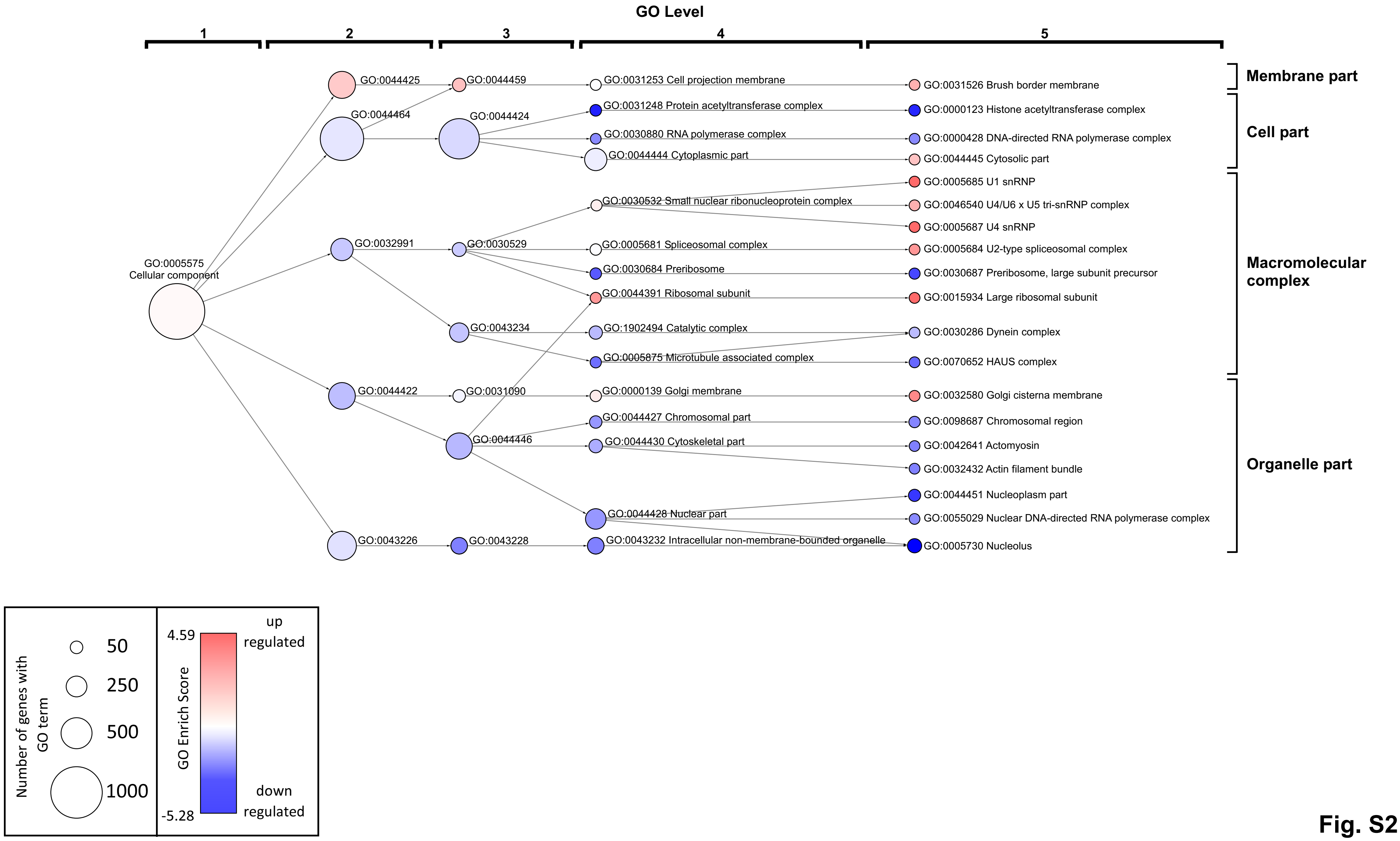

GO Level
1
2
3
4
5
Membrane part
Cell part
Macromolecular complex
Organelle part
GO:0005575 Cellular component
GO:0044425
GO:0044464
GO:0032991
GO:0044422
GO:0043226
GO:0044459
GO:0044424
GO:0030529
GO:0043234
GO:0031090
GO:0044446
GO:0043228
GO:0031253 Cell projection membrane
GO:0031248 Protein acetyltransferase complex
GO:0030880 RNA polymerase complex
GO:0044444 Cytoplasmic part
GO:0030532 Small nuclear ribonucleoprotein complex
GO:0005681 Spliceosomal complex
GO:0030684 Preribosome
GO:0044391 Ribosomal subunit
GO:1902494 Catalytic complex
GO:0005875 Microtubule associated complex
GO:0000139 Golgi membrane
GO:0044427 Chromosomal part
GO:0044430 Cytoskeletal part
GO:0044428 Nuclear part
GO:0043232 Intracellular non-membrane-bounded organelle
GO:0031526 Brush border membrane
GO:0000123 Histone acetyltransferase complex
GO:0000428 DNA-directed RNA polymerase complex
GO:0044445 Cytosolic part
GO:0005685 U1 snRNP
GO:0046540 U4/U6 x U5 tri-snRNP complex
GO:0005687 U4 snRNP
GO:0005684 U2-type spliceosomal complex
GO:0030687 Preribosome, large subunit precursor
GO:0015934 Large ribosomal subunit
GO:0030286 Dynein complex
GO:0070652 HAUS complex
GO:0032580 Golgi cisterna membrane
GO:0098687 Chromosomal region
GO:0042641 Actomyosin
GO:0032432 Actin filament bundle
GO:0044451 Nucleoplasm part
GO:0055029 Nuclear DNA-directed RNA polymerase complex
GO:0005730 Nucleolus
Number of genes with GO term
50
250
500
1000
GO Enrich Score
4.59
-5.28
up regulated
down regulated

Fig. S2B

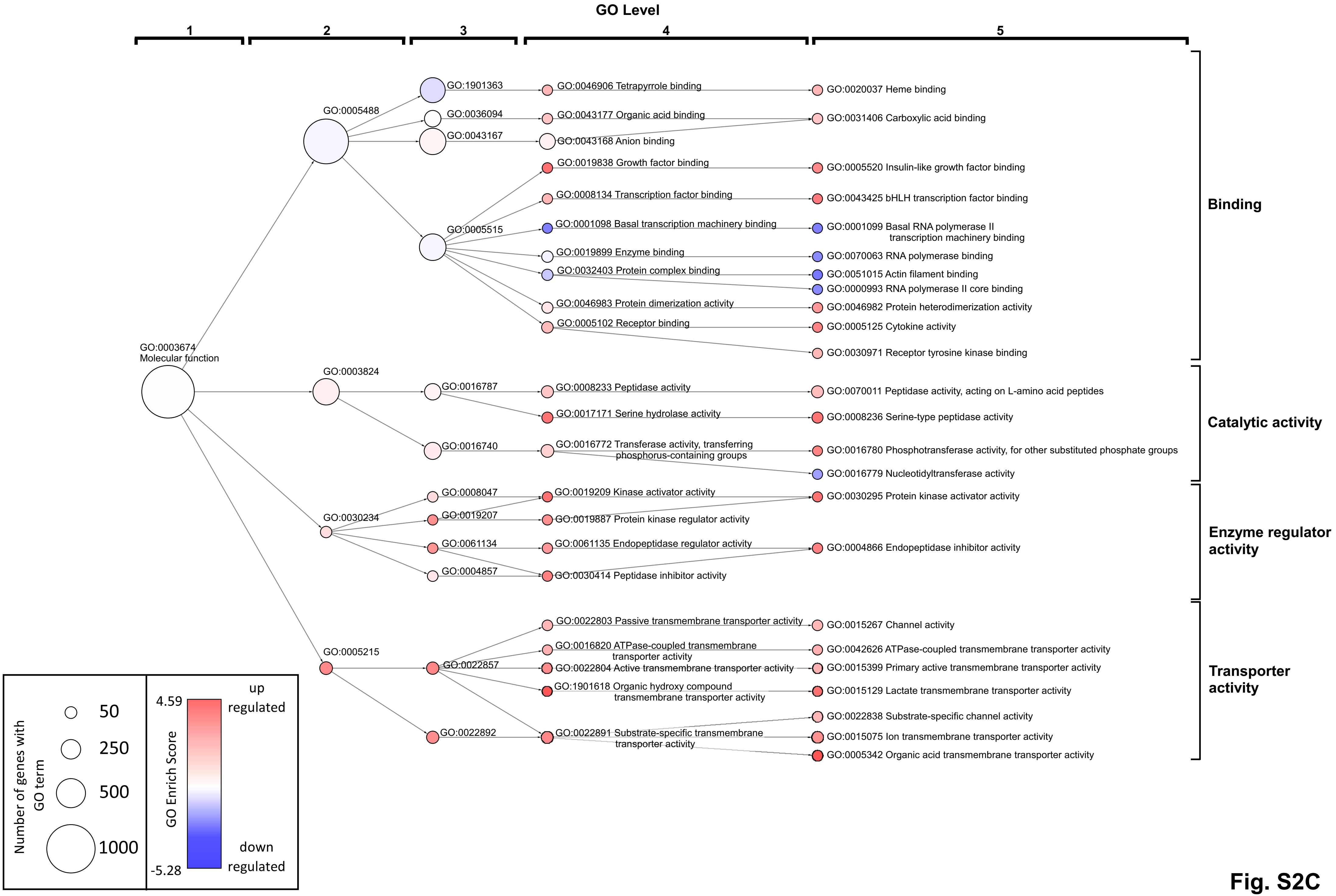

GO Level
1
2
3
4
5
GO:0003674
Molecular function
GO:0005488
GO:1901363
GO:0036094
GO:0043167
GO:0005515
GO:0046906 Tetrapyrrole binding
GO:0043177 Organic acid binding
GO:0043168 Anion binding
GO:0019838 Growth factor binding
GO:0008134 Transcription factor binding
GO:0001098 Basal transcription machinery binding
GO:0019899 Enzyme binding
GO:0032403 Protein complex binding
GO:0046983 Protein dimerization activity
GO:0005102 Receptor binding
GO:0020037 Heme binding
GO:0031406 Carboxylic acid binding
GO:0005520 Insulin-like growth factor binding
GO:0043425 bHLH transcription factor binding
GO:0001099 Basal RNA polymerase II transcription machinery binding
GO:0070063 RNA polymerase binding
GO:0051015 Actin filament binding
GO:0000993 RNA polymerase II core binding
GO:0046982 Protein heterodimerization activity
GO:0005125 Cytokine activity
GO:0030971 Receptor tyrosine kinase binding
Binding
GO:0003824
GO:0016787
GO:0016740
GO:0008233 Peptidase activity
GO:0017171 Serine hydrolase activity
GO:0016772 Transferase activity, transferring phosphorus-containing groups
GO:0070011 Peptidase activity, acting on L-amino acid peptides
GO:0008236 Serine-type peptidase activity
GO:0016780 Phosphotransferase activity, for other substituted phosphate groups
GO:0016779 Nucleotidyltransferase activity
Catalytic activity
GO:0030234
GO:0008047
GO:0019207
GO:0061134
GO:0004857
GO:0019209 Kinase activator activity
GO:0019887 Protein kinase regulator activity
GO:0061135 Endopeptidase regulator activity
GO:0030414 Peptidase inhibitor activity
GO:0030295 Protein kinase activator activity
GO:0004866 Endopeptidase inhibitor activity
Enzyme regulator activity
GO:0005215
GO:0022857
GO:0022892
GO:0022803 Passive transmembrane transporter activity
GO:0016820 ATPase-coupled transmembrane transporter activity
GO:0022804 Active transmembrane transporter activity
GO:1901618 Organic hydroxy compound transmembrane transporter activity
GO:0022891 Substrate-specific transmembrane transporter activity
GO:0015267 Channel activity
GO:0042626 ATPase-coupled transmembrane transporter activity
GO:0015399 Primary active transmembrane transporter activity
GO:0015129 Lactate transmembrane transporter activity
GO:0022838 Substrate-specific channel activity
GO:0015075 Ion transmembrane transporter activity
GO:0005342 Organic acid transmembrane transporter activity
Transporter activity
Number of genes with GO term
50
250
500
1000
GO Enrich Score
4.59
-5.28
up regulated
down regulated

Fig. S2C

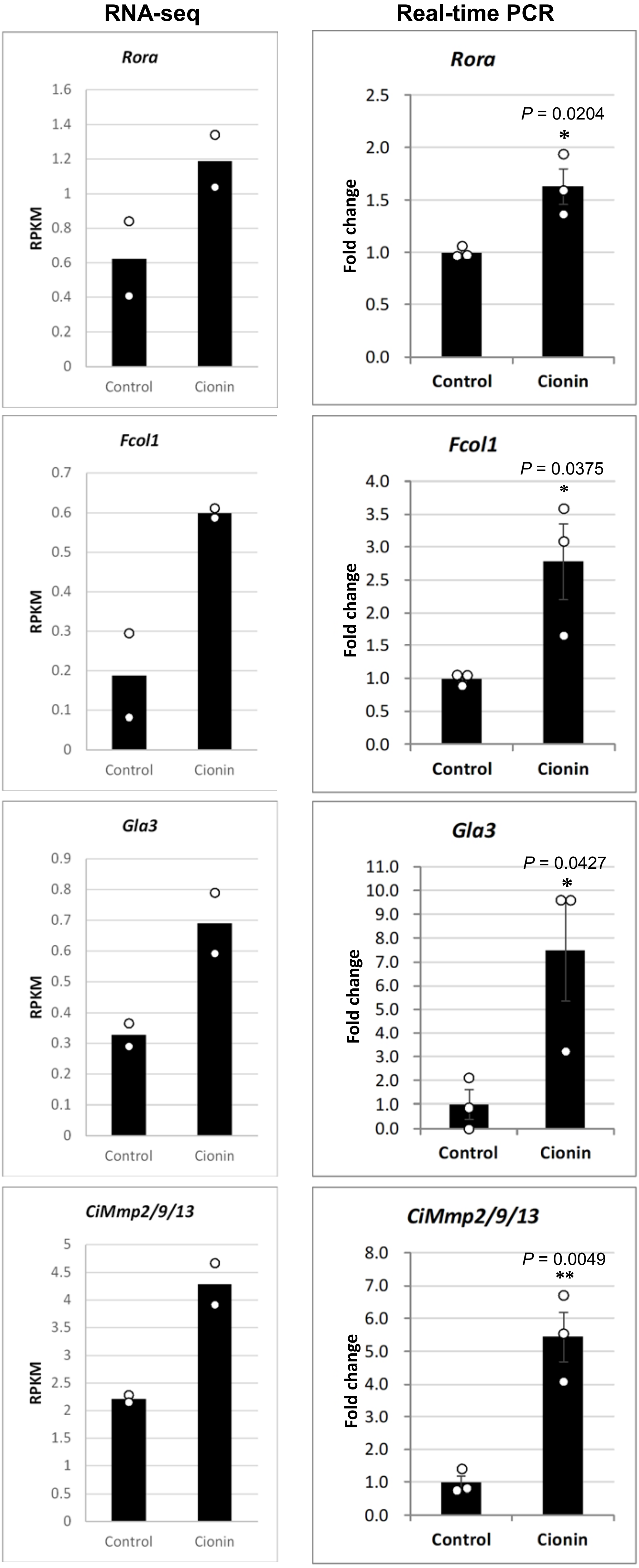

RNA-seq
Real-time PCR
Rora
Fcol1
Gla3
CiMmp2/9/13
RPKM
Fold change
Control
Cionin
P = 0.0204
*
P = 0.0375
*
P = 0.0427
*
P = 0.0049
**

Fig. S3

Table S1. Effects of RTK inhibitors on cionin-induced ovulation.

Reagent	Ovulation rate
Control	13.0%
Cionin	53.3%
Amuvatinib + cionin	57.1%
MGCD-265 + cionin	50.0%
Sunitinib malate + cionin	14.3%
Vandetanib + cionin	- (Follicles were collapsed)

14 or 15 follicles were tested for each reagent.

Concentration of cionin is 1 μM and RTK inhibitors are 1 mM, respectively.

Table S2. Expression of *Rora*, *Fcol1*, *Gla3*, and *CiMmp2/9/13 in RNA-seq* data and related GO terms

Gene name	Accession no.	Fold-change	Related GOs
Rora	KH.C8.101/ KY.Chr8.673	1.90	GO:0002009, GO:0046982, GO: 0046983, GO:0016772, GO:0043168
Fcol1	KH.C7.633/ KY.Chr7.425	2.11	GO:0048583, GO:0001503, GO:0050877, GO:0006909, GO:0019838
Gla3b	KH.C10.8/ KY.Chr10.1028	3.18	GO:0007599, GO:0051128, GO:0040012, GO:0051239, GO:0002682, GO:0048583, GO:0031667, GO:0007166, GO:0031669, GO:0009267, GO:0035690, GO:1901654, GO:0006935, GO:0007596, GO:0006812, GO:0044444, GO:0015075, GO:0005102, GO:0004866, GO:0070011, GO:0043168, GO:0022838, GO:0030414, GO0019209, GO:0015267, GO:0008233, GO:0022803, GO:0061135, GO:0030295
CiMmp2/9/13	KH.L76.4/ KY.Chr3.680	1.93	GO:0002682, GO:0048583, GO:0007165, GO:0007166, GO:0070887, GO:1901700, GO:1901698, GO:0001503, GO:0044712, GO:0019538, GO:0044444, GO:0070011, GO:0008233, GO:0008236, GO:0017171

Ciona Ghost Databases (http://ghost.zool.kyoto-u.ac.jp/cgi-bin/gb2/gbrowse/kh/ and http://ghost.zool.kyoto-u.ac.jp/default_ht.html) are referred to for the accession numbers for *Rora*, *Fcol1*, *Gla3b*, and *CiMmp2/9/13*. Level 4 and 5 GO terms are shown. Ratios of RPKM values of cionin-treated follicles to control follicles are shown as fold-changes.
